# Structure-activity relationship of 7-aryl-2-anilino-pyrrolopyrimidines as Mer and Axl tyrosine kinase inhibitors

**DOI:** 10.1080/14756366.2020.1825407

**Published:** 2020-09-24

**Authors:** Shin Hyuck Chung, Jiwon Park, Jung Wuk Lee, Jiho Song, Danbee Jung, Kyung Hoon Min

**Affiliations:** College of Pharmacy, Chung-Ang University, Seoul, Republic of Korea

**Keywords:** TAM familty, MER, Axl, pyrrolopyrimidine, kinase inhibitor

## Abstract

The TAM (Axl, Mer, and Tyro3) family is implicated in the survival and chemoresistance of tumours and has emerged as a potential therapeutic target. A novel series of 7-aryl-2-anilino-pyrrolopyrimidines were identified as potent Axl/Mer tyrosine kinase inhibitors without significant inhibition of Tyro3. A representative compound **27** exhibited IC_50_ values of 2 nM and 16 nM for Mer and Axl, respectively, and considerable inhibition for Mer phosphorylation in cells. Docking studies suggested that the formation of a salt bridge between the nitrogen of the aniline moiety with ASP678 of the Mer kinase domain as well as an interaction with the hinge region that most kinase inhibitors have in common would be essential to retain activity. These results could provide useful information for finding promising inhibitors of Axl/Mer for the treatment of cancer.

## Introduction

The TAM (TYRO3-AXL-MER) family consists of three receptor tyrosine kinases, Axl, Mer, and Tyro3. Several endogenous ligands have been identified for TAM receptors^1^. GAS6 binds to all three receptors but has a higher affinity for Axl compared to Mer and Tyro3. Protein S is known to be a specific ligand for Mer and Tyro3. TAM receptors are widely distributed in many tissues, including the nervous system, and they are involved in cell proliferation, survival, and migration as well as immune responses.

Oncogenic TAM receptor signalling is involved in tumour development^1^. Particularly, ectopic expression of TAM receptors has been associated with a poor prognosis in a variety of cancers^1^. Furthermore, it has been demonstrated that blockage of TAM signalling could improve the effectiveness of immunotherapy for cancer treatment^2^. TAM receptors, mainly the Mer receptor, induce M2 polarisation of macrophages in tumour microenvironments, which promotes tumour progression^3^.

Recent studies have demonstrated that Axl and Mer are implicated in resistance to chemotherapy and targeted therapy^4^^,^^5^. Thus, Axl/Mer inhibitors could provide a significant benefit for the treatment of patients with acquired resistance. With regard to a role of Mer in tumour associated macrophages, radiation therapy induced the upregulation of Mer in macrophages without changing the expression of Axl and tyro3^6^. *Mertk* knockout mice showed better overall survival than wild type mice after radiation therapy. Therefore, the Mer tyrosine kinase could be a target to prevent the resistance of tumours to radiation therapy.

**Figure 1.**
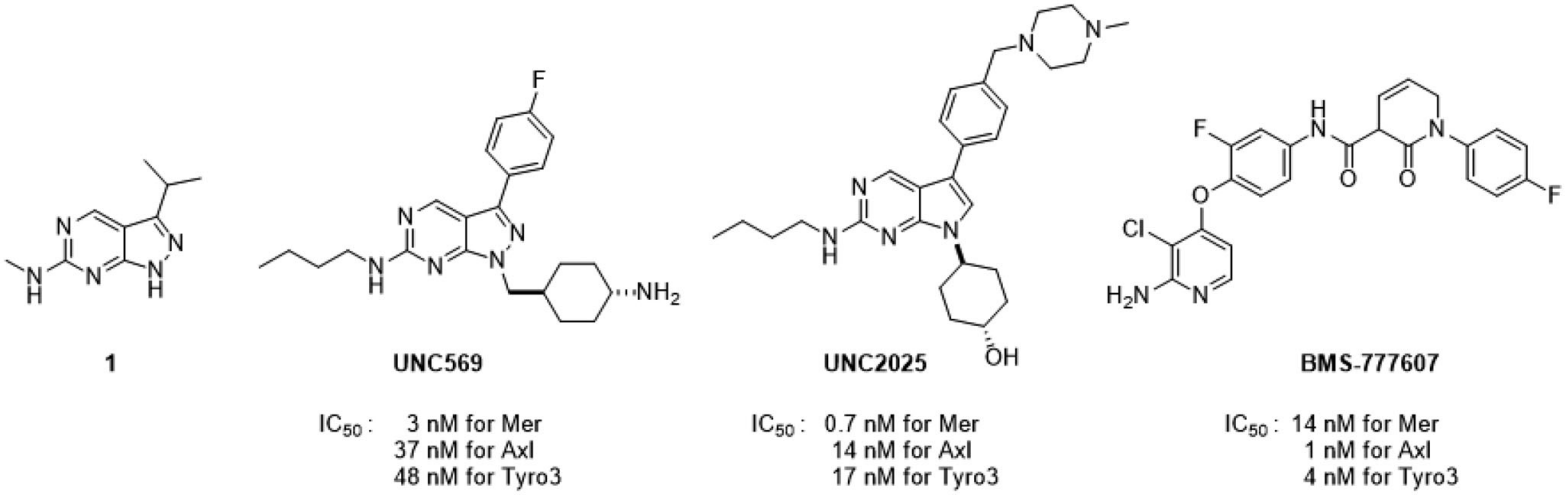
Structures and IC_50_ values of TAM kinase inhibitors.

Recent studies revealed that Axl is a key molecule in hematological malignancies including multiple myeloma^7^ and metastatic breast cancer^8^. The combination of a pan-TAM kinase inhibitor, BMS-777607, with anti-PD1 resulted in a better anti-tumour effect than each monotherapy alone in a mouse model^9^. Currently, many inhibitors for multiple TAM receptors are under clinical or preclinical investigation^10^. Representative TAM kinase inhibitors are shown in Figure 1.

Pyrazolopyrimidine **UNC569** was derived from an analysis of the co-crystal structure of **1** with Mer tyrosine kinase^11^ and showed potent inhibitory activity against the TAM family. Pyrrolopyrimidine **UNC2025** showed more potent inhibitory activity against Mer than **UNC569**, but both exhibited strong activity against Tyro3. The MET tyrosine kinase inhibitor, BMS-777607, also showed activity as a pan- TAM inhibitor.

Basically, the development of inhibitors specific to a single TAM receptor would be difficult because of structural similarities among the tree TAM receptors. However, Tyro3 is widely expressed in the adult central nervous system (CNS)^12^. Especially, Tyro3 is distributed in the nervous system at higher levels than Mer and Axl, indicating that inhibition of tyro3 could potentially lead to a toxicity issue even though Tyro3 could also be a therapeutic target for cancer. Mer is associated with resistance induced by Axl inhibition. Therefore, for the development of TAM kinase inhibitors, Axl/Mer inhibitors could provide an advantage over pan-TAM inhibitors. Moreover, the activation of Tyro3 could suppress retinal degeneration associated with Mer inhibition^13^. Therefore, it could be a plausible hypothesis that the discovery of Axl/Mer inhibitors that do not affect Tyro3 could give a better toxicity profile. Herein, we describe the identification of novel small-molecule inhibitors for Mer and Axl, and an investigation of their structure-activity relationship.

## Materials and methods

### Chemistry

All commercially available reagents were purchased from Sigma Aldrich®, Alfa Aesar, Tokyo Chemical Industry, Combi Blocks, Ark Pharm, Inc., or AstaTech. USP-grade solvents were purchased from Samchun Pure Chemical. HPLC grade solvents were purchased from either Fisher Scientific or J.T. Baker^®^. Microwave irradiation was performed using an Anton Paar Monoave 300. All reactions were monitored by thin-layer chromatography (TLC), using silica gel 60F_254_ from Merck and UV light visualisation. Flash chromatography was performed by Combiflash Rf+ (Teledyne Isco, USA) using silica gel (ZEOprep 60, 4063 µm, Zeochem LLC, USA) manually, a prepacked flash column Welux™ Column ultra-pure silica gel 4063 µm 60 Å (Intertechnologies Co., Ltd., Republic of Korea), or a RediSep^®^ Rf Gold (Teledyne Isco, USA). ^1^H and ^13^C^-^NMR spectra were obtained using Jeol Resonance ECZ 600 R (600 MHz) or Varian Gemini 2000 (300 Mhz). Chemical shifts were reported in parts per million (ppm, δ) using tetramethylsilane (TMS) as the internal standard. Coupling constants (J) were provided in Hertz (Hz). Splitting patterns were described as follows: s, singlet; d, doublet; t, triplet; q, quartette; p, pentet; dd, doublet of doublets; dt, doublet of triplets; td, triplet of doublets; m, multiplet; br, broad signal. High-resolution mass spectra (HRMS) were obtained using a Q Exative™ Hybrid Quadropole-Orbitrap Mass Spectrometer (Thermo Scientific) with the ESI method.

### N*-(3-Methoxyphenyl)-7–(4-methoxyphenyl)-7*H*-pyrrolo[2,3-d]pyrimidin-2-amine (2)*

A mixture of 3-methoxy aniline (11.18 µL 0.1 mmol), **7** (26 mg, 0.1 mmol), BINAP (2.4 mg, 0.004 mmol), Pd_2_(dba)_3_ (1.8 mg, 0.002 mmol), caesium carbonate (65 mg, 0.2 mmol) in anhydrous dioxane (2 ml) was stirred at 100 °C for 8 h. After being cooled to room temperature, the reaction mixture was filtered through celite. The filtrate was concentrated *in vacuo* and purified by MPLC with dichloromethane/methanol gave to **2** (11 mg, 32%).

^1^H-NMR (600 MHz, CDCl_3_) δ 8.71 (s, 1H), 7.62–7.64 (m, 3H), 7.28 (s, 1H), 7.16–7.19 (m, 2H), 7.03–7.04 (m, 3H), 6.52–6.55 (m, 2H), 3.88 (s, 3H), 3.70 (s, 3H); ^13^C-NMR (150 MHz, CDCl_3_) δ 160.25, 158.24, 156.10, 151.71, 150.98, 141.62, 130.82, 129.38, 126.31, 125.34, 114.44, 113.36, 110.67, 107.29, 103.85, 101.18, 55.57, 55.17. IR(neat): 2954, 2835, 1597, 1568, 1518, 1455, 1417, 1248, 1210, 1173, 1156, 1032, 832, 751, 733, 690 cm^−1^.

### *7–(4-Methoxyphenyl)-*N*-phenyl-7H-pyrrolo[2,3-d]pyrimidin-2-amine (3)*

Following the procedure for **2**, aniline and **7** provided the title compound 3 (12 mg, 39%).

^1^H-NMR (600 MHz, CDCl_3_) δ 8.70 (s, 1H), 7.65–7.70 (m, 4H), 7.29 (t, *J* = 7.9 Hz, 2H), 7.18–7.20 (m, 2H), 7.04 (dd, *J* = 6.9, 2.1 Hz, 2H), 6.97 (t, *J* = 7.6 Hz, 1H), 6.54 (d, *J* = 4.1 Hz, 1H), 3.88 (s, 3H); ^13^C-NMR (150 MHz, CDCl_3_) δ 158.18, 156.17, 151.72, 150.97, 140.36, 130.88, 128.83, 126.08, 124.98, 121.54, 118.37, 114.40, 113.45, 101.26, 55.61. IR(neat): 2954, 2835, 1597, 1568, 1536, 1518, 1483, 1417, 1210, 1156, 1032, 832, 766, 751, 733, 690, 596 cm^−1^.

### N*-([1,1′-Biphenyl]-3-yl)-7–(4-methoxyphenyl)-7*H*-pyrrolo[2,3-d]pyrimidin-2-amine (4)*

Following the procedure for **2,** [1,1′-biphenyl]-3-amine and **7** provided the title compound **4** (10 mg, 5%).

^1^H-NMR (600 MHz, CDCl_3_) δ 8.73 (s, 1H), 8.23 (t, *J* = 1.9 Hz, 1H), 7.61 (dd, *J* = 6.7, 2.2 Hz, 2H), 7.54–7.55 (m, 2H), 7.39–7.42 (m, 3H), 7.34–7.37 (m, 2H), 7.28 (s, 1H), 7.20–7.22 (m, 2H), 6.86 (dd, *J* = 6.7, 2.2 Hz, 2H), 6.56 (d, *J* = 3.8 Hz, 1H), 3.79 (s, 3H); ^13^C-NMR (150 MHz, CDCl_3_) δ 158.07, 156.12, 151.58, 151.08, 141.94, 141.47, 140.66, 130.74, 129.10, 128.64, 127.30, 127.15, 126.25, 125.00, 120.51, 117.29, 116.99, 114.47, 113.45, 101.23, 55.55. IR(neat): 2954, 2835, 1597, 1568, 1536, 1518, 1483, 1455, 1417, 1283, 1210, 1156, 1032, 832, 766, 751, 733, 690 cm^−1^.

### *7–(4-Methoxyphenyl)-2–(4-methylpiperazin-1-yl)-7*H*-pyrrolo[2,3-d]pyrimidine (5)*

A mixture of 1-methylpiperazine (11.1 µL, 0.1 mmol), **7** (26.1 mg, 0.1 mmol), and 4 M HCl in dioxane (50.0 µL, 0.2 mmol) in isopropanol (2 ml) was heated at 160 °C for 1 h in a microwave reactor. After concentration, the crude mixture was diluted with dichloromethane (100 ml) and washed with saturated NaHCO_3_ solution (10 ml) and water. After drying over MgSO_4_, the organic layer was concentrated *in vacuo* and purified by MPLC with dichloromethane/methanol to give the title compound **5** (5 mg, 15%).

^1^H-NMR (300 MHz, CDCl_3_) *δ* 8.63 (s, 1H), 7.64 (d, *J* = 9 Hz, 2H), 7.12 (d, *J* = 3.9 Hz, 1H), 7.02 (d, *J* = 9 Hz, 2H), 6.47 (d, *J* = 3.9 Hz, 1H), 3.87 (br, 7H), 2.51 (t, *J* = 5.1 Hz, 4H), 2.36 (s, 3H); ^13^C-NMR (150 MHz, CDCl_3_) *δ* 159.2, 157.7, 152.5, 150.5, 131.3, 125.2, 124.5, 114.3, 111.9, 101.0, 55.6, 55.1, 46.2, 44.3; IR(neat): 2934, 2840, 2792, 1607, 1551, 1524, 1508, 1431, 1386, 1362, 1330, 1248, 1227, 1203, 1170, 1143, 1034, 1006, 950, 830, 734 cm^−1^. HRMS (ESI): *m/z* calculated for C_18_H_21_N_5_O [M + H]^+^ 324.1819 found 324.1823.

### *2-Chloro-7–(4-methoxyphenyl)-7*H*-pyrrolo[2,3-d]pyrimidine (7)*

A mixture of 2-chloro-7H-pyrrolo[2,3-d]pyrimidine **6** (768 mg, 5.0 mmol), 4-methoxy phenyl boronic acid(1519 mg, 10 mmol), anhydrous pyridine (811 µL, 10 mmol), Copper(II)acetate (1362 mg, 7.5 mmol) in dichloromethane (10 ml) was stirred at room temperature for 12 h. The mixture was filtered through celite. The filtrate was concentrated *in vacuo* and purified by MPLC with dichloromethane/methanol to give the title compound **7** (218 mg, 17%).

^1^H-NMR (300 MHz, CDCl_3_) *δ* 8.88 (s, 1H), 7.56 (d, *J* = 9 Hz, 2H), 7.44 (d, *J* = 3.6 Hz, 1H), 7.05 (d, *J* = 9 Hz, 2H), 6.70 (d, *J* = 3.6 Hz, 1H), 3.88 (s, 3H); ^13^C-NMR (150 MHz, CDCl_3_) *δ* 158.9, 154.1, 151.6, 151.3, 130.1, 129.6, 125.4, 118.3, 114.8, 101.0, 55.6.

### *2–(2-Chloro-7*H*-pyrrolo[2,3-d]pyrimidin-7-yl)thiazole (8)*

A mixture of 2-chloro-7*H*-pyrrolo[2,3-d]pyrimidine **6** (150 mg, 0.977 mmol), 2-bromothiazole (436.2 µL, 4.88 mmol), copper(I) iodide (1.9 mg, 0.01 mmol), tripotassium phosphate (414.6 mg, 1.95 mmol), and 1,2-*trans*-cyclohexanediamine (12.0 µL, 0.098 mmol) in toluene (1 ml) was stirred at 100 °C for 24 h, and then cooled down to room temperature. The reaction mixture was concentrated and diluted with dichloromethane (100 ml). The organic layer was washed with water and brine. After the mixture was dried over MgSO_4_, the organic layer was concentrated *in vacuo* and purified by MPLC with chloroform/acetonitrile to give the title compound **8** (51 mg, 22%).

^1^H-NMR (300 MHz, CDCl_3_) *δ* 8.90 (s, 1H), 8.25 (d, *J* = 3.9 Hz, 1H), 7.63 (d, *J* = 3.3 Hz, 1H), 7.29 (d, *J* = 3.3 Hz, 1H), 6.77 (d, *J* = 3.9 Hz, 1H); ^13^C-NMR (150 MHz, CDCl_3_) *δ* 155.3, 154.6, 151.4, 150.7, 138.6, 127.6, 119.1, 116.8, 102.9.

### *5-Bromo-2-chloro-*N*-phenylpyrimidin-4-amine (10a)*

A mixture of 5-bromo-2,4-dichloropyrimidine **9** (683 mg, 3.0 mmol), aniline (273 µL, 3 mmol), and triethylamine (1254 µL, 9.0 mmol) in anhydrous isopropanol (10 ml) was stirred at room temperature for 12 h. The reaction mixture was diluted with dichloromethane (100 ml) and washed with saturated NaHCO_3_ solution (10 ml) and water. After drying over MgSO_4_, the organic layer was concentrated *in vacuo* and purified by MPLC with dichloromethane/methanol to give the title compound **10a** (584 mg, 68%).

^1^H-NMR (600 MHz, CDCl_3_) δ 8.28 (s, 1H), 7.60 (d, *J* = 8.3 Hz, 2H), 7.45–7.34 (m, 2H), 7.27 (d, *J* = 13.9 Hz, 1H), 7.23–7.16 (m, 1H); ^13^C-NMR (150 MHz, CDCl_3_) δ 159.24, 157.59, 157.16, 137.00, 129.27, 125.27, 121.30, 103.78.

### *5-Bromo-2-chloro-*N*-(4-(trifluoromethoxy)phenyl)pyrimidin-4-amine (10 b)*

Following the procedure for **10a**, 4-(trifluoromethoxy)aniline and **9** provided the title compound 10 b (89%).

^1^H-NMR (600 MHz, CDCl_3_) δ 8.31 (s, 1H), 7.65 (d, *J* = 9.0 Hz, 2H), 7.31 (s, 1H), 7.26 (s, 1H), 7.24 (s, 1H); ^13^C-NMR (151 MHz, CDCl_3_) δ 159.20, 157.86, 157.04, 145.99, 135.63, 122.50, 121.91, 120.53(q, *J* = 257 Hz), 103.775.

### Trans*-4-((5-Bromo-2-chloropyrimidin-4-yl)amino)cyclohexanol (10c)*

Following the procedure for **10a**, *trans*-4-Aminocyclohexanol and **9** provided the title compound 10c (97%).

^1^H-NMR (600 MHz, CDCl_3_) δ 8.11 (s, 1H), 5.31 (d, *J* = 6.9 Hz, 1H), 3.98–4.05 (m, 1H), 3.66–3.71 (m, 1H), 2.13–2.15 (m, 2H), 2.03–2.06 (m, 2H), 1.71 (s, 1H), 1.50 (ddd, *J* = 23.4, 13.1, 3.4 Hz, 2H), 1.30–1.37 (m, 2H); ^13^C-NMR (150 MHz, CDCl_3_) δ 159.42, 158.74, 156.38, 102.96, 69.60, 49.27, 33.67, 30.43.

### Trans*-4-((2-Chloro-5-((trimethylsilyl)ethynyl)pyrimidin-4-yl)amino)cyclohexanol (11c)*

A mixture of *trans*-4-((5-Bromo-2-chloropyrimidin-4-yl)amino)cyclohexanol **10c** (153 mg, 0.5 mmol), TMS acetylene (71.7 µL, 0.5 mmol), TEA (246.5 µL, 2.5 mmol), Pd(PPh_3_)Cl_2_ (7 mg, 0.01 mmol), CuI (2 mg, 0.01 mmol) in anhydrous toluene (10 ml) was stirred at 80 °C for 4 h. After being cooled to room temperature, the reaction mixture was filtered through celite. The filtrate was concentrated *in vacuo* and purified by MPLC with dichloromethane/methanol to give the title compound **11c** (39 mg, 24%).

^1^H-NMR (600 MHz, CDCl_3_) δ 8.08 (s, 1H), 5.45 (d, *J* = 7.7 Hz, 1H), 3.99–4.03 (m, 1H), 3.68–3.73 (m, 1H), 2.15–2.17 (m, 2H), 2.01–2.04 (m, 2H), 1.84 (s, 1H), 1.51 (ddd, *J* = 23.3, 12.8, 3.3 Hz, 2H), 1.31 (ddd, *J* = 24.0, 12.8, 3.2 Hz, 2H), 0.27 (s, 9H); ^13^C-NMR (150 MHz, CDCl_3_) δ 162.21, 159.71, 158.21, 106.46, 101.18, 96.04, 69.69, 48.94, 33.72, 30.56.

### *2-Chloro-7-phenyl-7*H*-pyrrolo[2,3-d]pyrimidine (12a)*

A mixture of **10a** (584 mg, 2.05 mmol), TMS acetylene (291 µL, 2.05 mmol), (1420 µL, 10.25 mmol) Pd(PPh_3_)Cl_2_ (28.7 mg, 0.041 mmol) and CuI (7.8 mg, 0.041 mmol) in anhydrous toluene (5 ml) was stirred at 80 °C for 4 h. After being cooled to room temperature, the reaction mixture was filtered through celite. The filtrate was concentrated *in vacuo* and purified by MPLC with dichloromethane/methanol to give **11a**. Next, a mixture of **11a** (607 mg, 2.01 mmol), TBAF 1 M in THF (4.02 ml, 4.02 mmol) in THF (25 ml) was stirred at 60 °C for 12 h. After the mixture was filtered through celite, the mixture was diluted with dichloromethane (100 ml) and washed with saturated NaHCO_3_ solution (10 ml) and brine. After drying over Na_2_SO_4_, the organic layer was concentrated *in vacuo* and purified by MPLC with dichloromethane/methanol to give the title compound **12a** (236 mg, 52%).

^1^H-NMR (600 MHz, CDCl_3_) δ 8.88 (s, 1H), 7.69 (dd, *J* = 8.5, 0.9 Hz, 2H), 7.51–7.55 (m, 3H), 7.40 (t, *J* = 7.5 Hz, 1H), 6.73 (d, *J* = 3.7 Hz, 1H); ^13^C-NMR (150 MHz, CDCl_3_) δ 154.34, 151.70, 151.57, 136.77, 129.78, 129.72, 127.58, 123.94, 118.66, 101.54.

### *2-Chloro-7–(4-(trifluoromethoxy)phenyl)-7*H*-pyrrolo[2,3-d]pyrimidine (12b)*

Following the procedure for **12a,** TMS acetylene and **10b** provided the title compound **12b** (28%).

^1^H-NMR (600 MHz, CDCl_3_) δ 8.90 (s, 1H), 7.76 (dd, *J* = 7.1, 1.9 Hz, 2H), 7.50 (d, *J* = 3.8 Hz, 1H), 7.40 (d, *J* = 8.8 Hz, 2H), 6.76 (d, *J* = 3.6 Hz, 1H); ^13^C-NMR (150 MHz, CDCl_3_) δ 154.53, 151.78, 151.73, 148.01, 135.23, 129.34, 125.19, 122.29, 120.50(q, *J* = 258.2 Hz, 118.64, 102.05.

### Trans*-4–(2-Chloro-7*H*-pyrrolo[2,3-d]pyrimidin-7-yl)cyclohexanol (12c)*

A mixture of *trans*-4-((2-Chloro-5-((trimethylsilyl)ethynyl)pyrimidin-4-yl)amino)cyclohexanol **11c** (38.7 mg, 0.12 mmol), TBAF 1 M in THF (240 µL, 0.24 mmol). in THF (10 ml) was stirred at 60 °C for 24 h. After the mixture was filtered through celite, the mixture was diluted with dichloromethane (100 ml) and washed with saturated NaHCO_3_ solution (10 ml) and brine. After drying over Na_2_SO_4_, the organic layer was concentrated *in vacuo* and purified by MPLC with dichloromethane/methanol to give the title compound **12c** (25 mg, 83%).

^1^H-NMR (600 MHz, CDCl_3_) δ 8.78 (s, 1H), 7.27 (s, 1H), 6.57 (d, *J* = 3.7 Hz, 1H), 4.72–4.76 (m, 1H), 3.77–3.81 (m, 1H), 2.10–2.18 (m, 4H), 1.82–1.89 (m, 3H), 1.62 (ddd, *J* = 24.2, 13.1, 3.3 Hz, 2H); ^13^C-NMR (150 MHz, CDCl_3_) δ 153.30, 151.62, 150.96, 126.61, 117.96, 100.25, 69.71, 52.52, 34.41, 31.05.

### 1–(2-Methoxy-4-nitrophenyl)-4-methylpiperazine (14a)

A mixture of 2-bromo-5-nitroanisole 13 (300 mg, 1.3 mmol), 1-methylpiperazine (199 µL, 1.8 mmol), BINAP (96.5 mg, 0.16 mmol) and Pd_2_(dba)_3_ (34.8 mg, 0.04 mmol), caesium carbonate (65 mg, 0.2 mmol) in anhydrous toluene (10 ml) was stirred at 120 °C for overnight. After being cooled to room temperature, the reaction mixture was filtered through celite. The filtrate was concentrated *in vacuo* and purified by MPLC with dichloromethane/methanol to give the title compound **14a** (232 mg, 71%).

^1^H-NMR (600 MHz, CDCl_3_) δ 7.86 (dd, *J* = 8.8, 2.5 Hz, 1H), 7.71 (d, *J* = 2.5 Hz, 1H), 6.90 (d, *J* = 8.8 Hz, 1H), 3.95 (s, 3H), 3.27 (s, 4H), 2.61 (s, 4H), 2.37 (s, 3H); ^13^C-NMR (150 MHz, CDCl_3_) δ 151.20, 147.38, 142.02, 117.83, 116.72, 106.54, 55.96, 54.99, 49.90, 46.13. HRMS (ESI): *m/z* calcd for C_12_H_18_N_3_O_3_ [M + H] + 252.1343, found 252.13525.

### N*-(2-Methoxy-4-nitrophenyl)-1-methylpiperidin-4-amine (14b)*

Following the procedure for **14a**, 4-amino-1-methylpiperidine and **13** provided the title compound **14b** (40%).

^1^H-NMR (300 MHz, CDCl_3_) *δ* 7.89 (dd, *J* = 2.4, 9 Hz, 1H), 7.62 (d, *J* = 2.4 Hz, 1H), 6.50 (d, *J* = 9 Hz, 1H), 4.96 (d, *J* = 7.8 Hz, 1H), 3.94 (s, 3H), 3.42 (br, 1H), 2.88 (d, *J* = 11.4 Hz, 2H), 2.35 (s, 3H), 2.21 (t, *J* = 11.4 Hz, 2H), 2.02–2.10 (m, 2H), 1.59–1.70 (m, 2H); ^13^C-NMR (150 MHz, CDCl_3_) *δ* 145.1, 143.1, 136.8, 119.9, 106.7, 104.9, 55.9, 54.1, 48.7, 46.0, 31.8.

### *1–(2-Methoxy-4-nitrophenyl)-*N,N*-dimethylpyrrolidin-3-amine (14c)*

Following the procedure for **14a**, 3-dimethylaminopyrrolidine and **13** provided the title compound **14c** (29%).

^1^H-NMR (600 MHz, CDCl_3_) δ 7.83 (dd, *J* = 9.0, 2.5 Hz, 1H), 7.65 (d, *J* = 2.5 Hz, 1H), 6.47 (d, *J* = 9.0 Hz, 1H), 3.85 (s, 3H), 3.72 (dd, *J* = 10.2, 7.0 Hz, 1H), 3.64–3.67 (m, 2H), 3.50 (dd, *J* = 10.1, 8.6 Hz, 1H), 2.76 (t, *J* = 9.0 Hz, 1H), 2.32 (s, 6H), 2.14–2.18 (m, 1H), 1.84–1.89 (m, 1H); ^13^C-NMR (150 MHz, CDCl_3_) δ 147.47, 144.79, 137.73, 119.47, 111.68, 107.35, 77.24, 77.03, 76.82, 65.21, 56.07, 55.26, 49.99, 44.32, 29.93.

### N*-(3-Methoxy-4–(4-methylpiperazin-1-yl)phenyl)-7–(4-methoxyphenyl)-7*H*-pyrrolo[2,3-d]pyrimidin-2-amine (16)*

A mixture of **14a** (25.1 mg, 0.1 mmol) and Pd/C (10 w/w%) in MeOH (10 ml) were sealed with a septum and substituted with H_2_ gas. The reaction mixture was stirred at room temperature for 4 h. The mixture was filtered off and the filtrate was concentrated *in vacuo* to give the corresponding amine **15a**. Successively, a mixture of **15a**, **7** (26.0 mg, 0.1 mmol), BINAP (9.3 mg, 0.0150 mmol), sodium *tert*-butoxide (19.2 mg, 0.2 mmol), and Pd_2_(dba)_3_ (4.6 mg, 0.005 mmol) in anhydrous toluene (4 ml) was stirred at 100 °C for 20 h. After being cooled to room temperature, the reaction mixture was concentrated and diluted with dichloromethane (100 ml). The organic layer was washed with water and brine. After the mixture was dried over MgSO_4_, the organic layer was concentrated *in vacuo* and purified by MPLC with chloroform/acetonitrile to give the title compound **16** (25 mg, 56%).

^1^H-NMR (300 MHz, CDCl_3_) *δ* 8.68 (s, 1H), 7.56–7.61 (m, 3H), 7.24 (br, 1H), 7.14 (d, *J* = 3.9 Hz, 1H), 6.98–7.04 (m, 2H), 6.93 (dd, *J* = 2.4, 8.7 Hz, 1H), 6.87 (d, *J* = 8.7 Hz, 1H), 6.53 (d, *J* = 3.6 Hz, 1H), 3.87 (s, 3H), 3.66 (s, 3H), 3.07 (br, 4H), 2.68 (br, 4H), 2.39 (s, 3H); ^13^C-NMR (150 MHz, CDCl_3_) *δ* 158.3, 156.3, 152.5, 151.9, 136.3, 135.4, 130.9, 126.4, 125.7, 118.4, 114.4, 113.0, 110.2, 102.8, 101.1, 55.6, 55.3(2 C), 50.7, 45.9; IR(neat): 2935, 2798, 1599, 1562, 1530, 1510, 1417, 1249, 1212, 1151, 1033, 1012, 832, 733 cm^−1^. HRMS (ESI): *m/z* calcd for C_25_H_28_N_6_O_2_ [M + H]^+^ 445.2347 found 445.2344.

### *2-Methoxy-N4-(7–(4-methoxyphenyl)-7*H*-pyrrolo[2,3-d]pyrimidin-2-yl)-*N*1-(1-methylpiperidin-4-yl)benzene-1,4-diamine (17)*

Following the procedure for **16**, **14b** and **7** provided the title compound **17** (17%).

^1^H-NMR (300 MHz, CDCl_3_) *δ* 8.65 (s, 1H), 7.60 (d, *J* = 9 Hz, 2H), 7.49 (d, *J* = 2.4 Hz, 1H), 7.12 (d, *J* = 3.6 Hz, 1H), 7.08 (s, 1H), 7.00 (d, *J* = 9 Hz, 2H), 6.84 (dd, *J* = 2.4, 8.4 Hz, 1H), 6.55 (d, *J* = 8.4 Hz, 1H), 6.51 (d, *J* = 3.6 Hz, 1H), 3.87 (s, 3H), 3.67 (s, 3H), 3.23–3.30 (m, 1H), 2.86 (d, *J* = 11.7 Hz, 2H), 2.34 (s, 3H), 2.20 (t, *J* = 10.8 Hz, 2H), 2.05–2.11 (m, 2H), 1.50–1.62 (m, 2H); IR(neat): 2933, 1601, 1563, 1513, 1416, 1248, 1210, 1034, 832, 732 cm^−1^. HRMS (ESI): *m/z* calcd for C_26_H_30_N_6_O_2_ [M + H]^+^ 459.2503 found 459.2490.

### N*-(4–(3-(Dimethylamino)pyrrolidin-1-yl)-3-methoxyphenyl)-7–(4-methoxyphenyl)-7*H*-pyrrolo[2,3-d]pyrimidin-2-amine (18)*

Following the procedure for **16**, **14c** and **7** provided the title compound **18** (19%).

^1^H-NMR (600 MHz, CDCl_3_) δ 8.68 (s, 1H), 7.65–7.54 (m, 3H), 7.26 (s, 3H), 7.18–6.97 (m, 4H), 6.89 (dd, *J* = 8.6, 2.4 Hz, 1H), 6.73 (d, *J* = 9.0 Hz, 1H), 6.53 (d, *J* = 3.4 Hz, 1H), 3.88 (s, 3H), 3.66 (s, 3H), 3.50–3.36 (1H), 3.36–3.24 (1H), 3.24–3.07 (1H), 2.43 (s, 6H), 2.26–2.14 (1H), 2.01–1.87 (1H); ^13^C-NMR (150 MHz, CDCl_3_) δ 158.30, 156.49, 151.99, 151.35, 151.02, 130.95, 126.18, 125.64, 123.54, 118.21, 116.22, 114.42, 112.97, 110.55, 103.48, 101.14, 65.15, 55.62, 55.42, 49.75, 43.18, 41.04; IR(neat): 2935, 2821, 1600, 1561, 1511, 1417, 1346, 1249, 1209, 1035, 962, 832, 748, 698 cm^−1^.

### 1–(3-Methoxy-5-nitrophenyl)-4-methylpiperazine (20a)

A mixture of 3-bromo-5-anisole **19** (232 mg, 1 mmol), 1-methylpiperazine (122 µL, 1.1 mmol), caesium carbonate (651.6 mg, 2 mmol), BINAP (37.4 mg, 0.06 mmol), and Pd_2_(dba)_3_ (18.3 mg, 0.02 mmol) in anhydrous toluene (2.5 ml) was stirred at 120 °C for overnight. After being cooled to room temperature, the reaction mixture was filtered through celite. The filtrate was concentrated *in vacuo* and purified by MPLC with dichloromethane/methanol to give the title compound **20a** (197 mg, 44%).

^1^H-NMR (600 MHz, CDCl_3_) δ 7.40 (t, *J* = 2.0 Hz, 1H), 7.19 (t, *J* = 2.0 Hz, 1H), 6.68 (t, *J* = 2.2 Hz, 1H), 3.85 (s, 3H), 3.28 (t, *J* = 5.0 Hz, 4H), 2.57 (t, *J* = 5.1 Hz, 4H), 2.36 (s, 3H); ^13^C-NMR (150 MHz, CDCl_3_) δ 160.85, 152.33, 150.05, 107.67, 103.53, 98.26, 55.76, 54.71, 48.27, 46.10. HRMS (ESI): *m/z* calcd for C_12_H_18_N_3_O_3_ [M + H] + 252.1343, found 252.13544.

### 1–(3-Methoxy-5-nitrophenyl)-4-(pyrrolidin-1-yl) piperidine (20b)

Following the procedure for **20a**, 4-(pyrrolidinyl) piperazine and **19** provided the title compound **20b** (75%).

^1^H-NMR (600 MHz, CDCl_3_) δ 7.40 (t, *J* = 2.1 Hz, 1H), 7.15 (t, *J* = 2.1 Hz, 1H), 6.69 (t, *J* = 2.3 Hz, 1H), 3.84 (s, 3H), 3.75–3.70 (2H), 2.92–2.83 (m, 2H), 2.65–2.55 (m, 4H), 2.18 (tt, *J* = 10.5, 3.8 Hz, 1H), 2.00 (d, *J* = 12.1 Hz, 2H), 1.86–1.75 (m, 4H), 1.71–1.59 (2H); ^13^C-NMR (150 MHz, CDCl_3_) δ 160.80, 152.28, 150.03, 107.85, 103.67, 97.73, 61.43, 55.72, 51.51, 47.54, 30.88, 23.24. HRMS (ESI): *m/z* calcd for C_16_H_24_N_3_O_3_ [M + H] + 306.1812, found 306.18291.

### 1–(3-Methoxy-5-nitrophenyl)-4-methyl-4-(pyrrolidin-1-yl) piperidine (20c)

Following the procedure for **20a**, 4-methyl-4-(pyrrolidin-1-yl)piperidine and **19** provided the title compound **20c** (67%).

^1^H-NMR (600 MHz, CDCl_3_) δ 7.41 (t, *J* = 2.1 Hz, 1H), 7.13 (t, *J* = 2.0 Hz, 1H), 6.69 (t, *J* = 2.2 Hz, 1H), 3.84 (s, 3H), 3.31–3.27 (m, 4H), 2.61 (s, 4H), 1.87 (d, *J* = 13.8 Hz, 2H), 1.74–1.72 (m, 4H), 1.59–1.55 (m, 2H), 0.98 (s, 3H); ^13^C-NMR (150 MHz, CDCl_3_) δ 160.77, 152.61, 150.04, 107.32, 103.30, 97.28, 55.70, 51.95, 44, 52, 44.49, 36.12, 24.10, 16.43. HRMS (ESI): *m/z* calcd for C_17_H_26_N_3_O_3_ [M + H] + 320.1969, found 320.19865.

### 1–(3-Methoxy-5-nitrophenyl)-3-(pyrrolidin-1-yl) piperidine (20d)

Following the procedure for **20a,** pyrrolidine and **19** provided the title compound **20d** (90%).

^1^H-NMR (600 MHz, CDCl_3_) δ 7.38 (t, *J* = 2.2 Hz, 1H), 7.16 (t, *J* = 2.1 Hz, 1H), 6.68 (t, *J* = 2.3 Hz, 1H), 3.92–3.86 (m, 1H), 3.85 (s, 3H), 3.65 (d, *J* = 12.6 Hz, 1H), 2.84 (td, *J* = 12.1, 2.9 Hz, 2H), 2.74 (s, 4H), 2.53–2.26 (m, 1H), 2.11 (d, *J* = 9.8 Hz, 1H), 1.93–1.79 (m, 5H), 1.71–1.61 (m, 1H); ^13^C-NMR (150 MHz, CDCl_3_) δ 160.89, 152.28, 150.12, 108.00, 103.88, 97.77, 60.52, 55.76, 51.59, 49.11, 23.55, 23.22. HRMS (ESI): *m/z* calcd for C_16_H_24_N_3_O_3_ [M + H]^+^ 306.1812, found 306.18253.

### 1–(3-Methoxy-5-nitrophenyl)-4-(oxetan-3-yl) piperazine (20e)

Following the procedure for **20a,** 1-oxetan-3-yl-piperazine and **19** provided the title compound **20e** (64%).

^1^H-NMR (600 MHz, CDCl_3_) δ 7.39 (t, *J* = 2.0 Hz, 1H), 7.20 (t, *J* = 1.9 Hz, 1H), 6.68 (t, *J* = 2.1 Hz, 1H), 4.71 (t, *J* = 6.6 Hz, 2H), 4.65 (t, *J* = 6.1 Hz, 2H), 3.86 (s, 3H), 3.62–3.49 (m, 1H), 3.30 (t, *J* = 5.0 Hz, 4H), 2.55–2.44 (4H); ^13^C-NMR (150 MHz, CDCl_3_) δ 160.91, 152.28, 150.09, 107.85, 103.63, 98.50, 75.44, 59.16, 55.84, 49.34, 48.09. HRMS (ESI): *m/z* calcd for C_14_H_20_N_3_O_4_ [M + H] + 294.1449, found 294.1461.

### 8–(3-Methoxy-5-nitrophenyl)-8-azaspiro[4.5]decane (20f)

Following the procedure for **20a,** 8-aza-sprio[4,5]decane and **19** provided the title compound **20f** (93%).

^1^H-NMR (600 MHz, CDCl_3_) δ 7.40 (t, *J* = 2.1 Hz, 1H), 7.14 (d, *J* = 4.0 Hz, 1H), 6.69 (t, *J* = 2.2 Hz, 1H), 3.84 (s, 3H), 3.24 (t, *J* = 5.7 Hz, 4H), 1.64–1.66 (m, 4H), 1.59 (t, *J* = 5.7 Hz, 4H), 1.46–1.48 (m, 4H); ^13^C-NMR (150 MHz, CDCl_3_) δ 160.88, 152.72, 150.14, 107.69, 103.64, 97.59, 55.78, 46.60, 40.77, 37.66, 36.92, 24.39.

### 8–(3-Methoxy-5-nitrophenyl)-1-oxa-8-azaspiro[4.5]decane (20g)

Following the procedure for **20a,** 1-oxs-8-aza-sprio[4,5]decane-HCl and **19** provided the title compound **20g** (89%).

^1^H-NMR (600 MHz, CDCl_3_) δ 7.39 (t, *J* = 2.1 Hz, 1H), 7.13 (t, *J* = 2.0 Hz, 1H), 6.67 (t, *J* = 2.2 Hz, 1H), 3.85 (q, *J* = 6.9 Hz, 5H), 3.31–3.38 (m, 4H), 1.93–1.96 (m, 2H), 1.70–1.73 (m, 6H); ^13^C-NMR (150 MHz, CDCl_3_) δ 160.93, 152.30, 150.18, 107.85, 103.83, 97.72, 79.59, 66.96, 55.79, 46.30, 36.67, 35.72, 25.53.

### 3-Methoxy-5-nitrobenzaldehyde (23)

A mixture of 3-methoxy-5-nitrobenzonitirle **22** (359 mg, 2.0 mmol) and 1.0 M diisobutyl aluminium hydride in THF (5.0 ml, 5.0 mmol) in anhydrous toluene (20 ml) was stirred at 0 °C for 3 h. The reaction mixture was concentrated and diluted with dichloromethane (100 ml). The organic layer was washed with water and brine. After the mixture was dried over Na_2_SO_4_, the organic layer was concentrated *in vacuo* and purified by MPLC with hexanes/ethyl acetate to give the title compound **23** (84 mg, 23%).

^1^H-NMR (600 MHz, CDCl_3_) δ 10.04 (s, 1H), 8.28 (s, 1H), 7.97 (t, *J* = 2.2 Hz, 1H), 7.71 (t, *J* = 1.2 Hz, 1H), 3.96 (s, 3H); ^13^C-NMR (150 MHz, CDCl_3_) δ 189.73, 160.94, 149.78, 138.30, 119.26, 117.17, 114.64, 56.46.

### 1–(3-Methoxy-5-nitrobenzyl)-4-methylpiperazine (24)

A mixture of **23** (83 mg, 0.458 mmol), 1-methylpiperazine (66 µL, 0.596 mmol), acetic acid (26 µL, 0.458 mmol), and NaBH(OAc)_3_ (291 mg, 1.375 mmol) in 1,2-dichloroethane (5 ml) was stirred at room temperature for 12 h. The mixture was diluted with dichloromethane (100 ml) and washed with saturated NaHCO_3_ solution (10 ml) and brine. After drying over Na_2_SO_4_, the organic layer was concentrated *in vacuo* and purified by MPLC with hexane/ethyl acetate to give the title compound **24** (40 mg, 33%).

1H-NMR (600 MHz, CDCl_3_) δ 7.82 (s, 1H), 7.61 (t, *J* = 2.1 Hz, 1H), 7.23 (s, 1H), 3.89 (s, 3H), 3.55 (s, 2H), 2.41 (d, *J* = 113.6 Hz, 11H); 13 C-NMR (150 MHz, CDCl_3_) δ 160.08, 149.26, 141.81, 121.49, 116.13, 106.77, 77.24, 77.03, 76.82, 62.03, 55.88, 55.03, 53.00, 45.97.

### N*-(3-Methoxy-5–(4-methylpiperazin-1-yl)phenyl)-7–(4-methoxyphenyl)-7*H*-pyrrolo[2,3-d]pyrimidin-2-amine (25)*

A mixture of **20a** (25.1 mg, 0.1 mmol) and Pd/C (10 w/w%) in MeOH (10 ml) was sealed with a septum and substituted with H_2_ gas. The reaction mixture was stirred at room temperature for 4 h. The mixture was filtered off and the filtrate was concentrated *in vacuo* to give compound **21a**. Successively, a mixture of **21a**, **7** (26.0 mg, 0.1 mmol), 4 M HCl in dioxane (50 µL, 0.2 mmol) in anhydrous isopropanol (2 ml) was heated at 160 °C for 1 h in a microwave reactor. After being cooled to room temperature, the reaction mixture was concentrated and diluted with dichloromethane (100 ml). The organic layer was washed with water and brine. After the mixture was dried over MgSO_4_, the organic layer was concentrated *in vacuo* and purified by MPLC with chloroform/acetonitrile to give the title compound **25** (13 mg, 29%).

^1^H-NMR (300 MHz, CDCl_3_) *δ* 8.69 (s, 1H), 7.59 (d, *J* = 9 Hz, 2H), 7.25 (s, 1H), 7.14 (d, *J* = 3.6 Hz, 1H), 7. 02 (d, *J* = 9 Hz, 2H), 6.99 (t, *J* = 2.1 Hz, 1H), 6.80 (t, *J* = 2.1 Hz, 1H), 6.54 (d, *J* = 3.6 Hz, 1H), 6.11 (t, *J* = 2.1 Hz, 1H), 3.88 (s, 3H), 3.70 (s, 3H), 3.10 (t, *J* = 5.1 Hz, 4H) 2.49 (t, *J* = 5.1 Hz, 4H), 2.34 (s, 3H); ^13^C-NMR (150 MHz, CDCl_3_) *δ* 160.9, 158.2, 156.2, 152.8, 151.7, 151.0, 142.0, 130.9, 126.5, 125.7, 114.5, 113.2, 101.1, 98.7, 96.2, 95.2, 55.5, 55.2, 55.1, 48.9, 46.1; IR(neat): 2934, 2837, 1593, 1568, 1536, 1518, 1483, 1452, 1418, 1248, 1209, 1161, 1032, 1003, 831, 734, 699 cm^−1^; HRMS (ESI): *m/z* calcd for C_25_H_28_N_6_O_2_ [M + H]^+^ 445.2347 found 445.2345.

### N*-(3-Methoxy-5-((4-methylpiperazin-1-yl)methyl)phenyl)-7–(4-methoxyphenyl)-7*H*-pyrrolo[2,3-d]pyrimidin-2-amine (26)*

Following the procedure for **25, 24** and **7** provided the title compound **26** (51%).

^1^H-NMR (300 MHz, CDCl_3_) *δ* 8.69 (s, 1H), 7.59 (d, *J* = 9 Hz, 2H), 7.25 (s, 1H), 7.14 (d, *J* = 3.6 Hz, 1H), 7. 02 (d, *J* = 9 Hz, 2H), 6.99 (t, *J* = 2.1 Hz, 1H), 6.80 (t, *J* = 2.1 Hz, 1H), 6.54 (d, *J* = 3.6 Hz, 1H), 6.11 (t, *J* = 2.1 Hz, 1H), 3.88 (s, 3H), 3.70 (s, 3H), 3.10 (t, *J* = 5.1 Hz, 4H) 2.49 (t, *J* = 5.1 Hz, 4H), 2.34 (s, 3H); ^13^C-NMR (150 MHz, CDCl_3_) *δ* 160.9, 158.2, 156.2, 152.8, 151.7, 151.0, 142.0, 130.9, 126.5, 125.7, 114.5, 113.2, 101.1, 98.7, 96.2, 95.2, 55.5, 55.2, 55.1, 48.9, 46.1; IR(neat): 2934, 2836, 2800, 1599, 1569, 1537, 1518, 1456, 1417, 1349, 1248, 1209, 1160, 1064, 832, 734, 699 cm^−1^. HRMS (ESI): *m/z* calcd for C_25_H_28_N_6_O_2_ [M + H]^+^ 445.2347 found 445.2345.

### N*-(3-Methoxy-5–(4-(pyrrolidin-1-yl)piperidin-1-yl)phenyl)-7–(4-methoxyphenyl)-7*H*-pyrrolo[2,3-d]pyrimidin-2-amine (27)*

Following the procedure for **25, 20b** and **7** provided the title compound **27** (10%).

^1^H-NMR (300 MHz, CDCl_3_) *δ*: 8.69 (s, 1H), 7.60 (d, *J* = 9 Hz, 2H), 7.17 (s, 1H), 7.15 (d, *J* = 3.6 Hz, 1H), 7.02 (d, *J* = 9 Hz, 2H), 6.94 (t, *J* = 1.8 Hz, 1H), 6.81 (t, *J* = 1.8 Hz, 1H), 6.54 (d, *J* = 3.6 Hz, 1H), 6.12 (t, *J* = 1.8 Hz, 1H), 3.88 (s, 3H), 3.70 (s, 3H), 3.60 (d, *J* = 12.3 Hz, 2H), 2.80–2.89 (m, 4H), 2.62 (t, *J* = 12.3 Hz, 2H), 1.94–2.12 (m, 1H), 1.66–2.02 (m, 8H); ^13^C-NMR (150 MHz, CDCl_3_) *δ* 160.9, 158.3, 156.1, 152.1, 151.7, 150.9, 142.0, 131.0, 126.7, 125.6, 114.6, 113.2, 101.2, 99.4, 97.3, 95.9, 62.0, 55.9, 55.2, 50.2, 48.5, 27.5, 23.6; IR(neat): 2954, 1592, 1568, 1537, 1519, 1418, 1248, 1209, 1034, 1157, 831, 734 cm^−1^. HRMS (ESI): *m/z* calcd for C_29_H_34_N_6_O_2_ [M + H]^+^ 499.2816 found 499.2812.

### N*-(3-Methoxy-5–(4-methyl-4-(pyrrolidin-1-yl)piperidin-1-yl)phenyl)-7–(4-methoxyphenyl)-7*H*-pyrrolo[2,3-d]pyrimidin-2-amine (28)*

Following the procedure for **25, 20c** and **7** provided the title compound **28** (22%).

^1^H-NMR (600 MHz, CDCl_3_) δ 8.70 (s, 1H), 7.61 (dd, *J* = 6.9, 2.0 Hz, 2H), 7.35 (s, 1H), 7.26 (s, 1H), 7.14 (d, *J* = 3.6 Hz, 1H), 7.01 (dd, *J* = 6.9, 1.9 Hz, 2H), 6.90 (s, 1H), 6.84 (d, *J* = 1.6 Hz, 1H), 6.52 (d, *J* = 3.6 Hz, 1H), 6.14 (t, *J* = 2.0 Hz, 1H), 3.85 (s, 3H), 3.69 (s, 3H), 3.18–3.22 (m, 2H), 3.01–3.05 (m, 2H), 2.60 (s, 4H), 1.76–1.80 (m, 2H), 1.72 (d, *J* = 5.9 Hz, 4H), 1.50–1.54 (m, 2H), 0.96 (s, 3H); ^13^C-NMR (150 MHz, CDCl_3_) δ 160.96, 158.27, 156.48, 153.26, 151.83, 151.06, 141.97, 131.00, 126.44, 125.64, 114.50, 113.17, 101.17, 98.75, 96.18, 94.59, 55.57, 55.24, 52.25, 45.23, 44.52, 36.24, 24.14, 16.50; IR(neat): 2958, 2835, 1603, 1569, 1536, 1519, 1484, 1458, 1419, 1349, 1248, 1211, 1156, 1071, 1034 cm^−1^.

### N*-(3-Methoxy-5–(3-(pyrrolidin-1-yl)piperidin-1-yl)phenyl)-7–(4-methoxyphenyl)-7*H*-pyrrolo[2,3-d]pyrimidin-2-amine (29)*

Following the procedure for **25, 20d** and **7** provided the title compound **29** (11%).

^1^H-NMR (600 MHz, CDCl_3_) δ 8.68 (s, 1H), 7.60–7.62 (m, 2H), 7.15 (d, *J* = 3.6 Hz, 1H), 7.10 (s, 1H), 7.01–7.03 (m, 2H), 6.98 (s, 1H), 6.76 (s, 1H), 6.53 (d, *J* = 3.7 Hz, 1H), 6.13 (t, *J* = 2.0 Hz, 1H), 3.86 (s, 3H), 3.84–3.77 (1H), 3.68 (s, 3H), 3.55–3.45 (1H), 2.63–2.66 (m, 5H), 2.32 (s, 1H), 2.06 (d, *J* = 12.5 Hz, 1H), 1.81 (s, 5H), 1.72 (d, *J* = 6.7 Hz, 2H), 1.59–1.67 (m, 1H); ^13^C-NMR (150 MHz, CDCl_3_) δ 161.14, 158.38, 156.44, 151.92, 151.00, 142.05, 131.07, 126.39, 125.83, 125.54, 114.61, 113.36, 101.17, 99.54, 97.05, 95.19, 60.95, 55.60, 55.36, 55.26, 51.66, 51.53, 49.90, 24.02, 23.33; IR(neat): 2937, 1594, 1567, 1537, 1519, 1484, 1461, 1418, 1356, 1249, 1207, 1162, 1030, 832, 735 cm^−1^.

### N*-(3-Methoxy-5–(4-(oxetan-3-yl)piperazin-1-yl)phenyl)-7–(4-methoxyphenyl)-7*H*-pyrrolo[2,3-d]pyrimidin-2-amine (30)*

Following the procedure for **25, 20e** and **7** provided the title compound **30** (27%).

^1^H-NMR (600 MHz, CDCl_3_) δ 8.70 (s, 1H), 7.89–7.76 (0H), 7.76–7.65 (0H), 7.59 (d, *J* = 8.7 Hz, 2H), 7.47–7.37 (0H), 7.34 (s, 1H), 7.25–7.19 (0H), 7.14 (d, *J* = 3.4 Hz, 1H), 7.07 (s, 1H), 7.01 (d, *J* = 8.7 Hz, 2H), 6.82–6.77 (0H), 6.73 (s, 1H), 6.53 (d, *J* = 3.4 Hz, 1H), 6.10 (s, 1H), 4.66–4.71 (m, 4H), 3.88 (d, *J* = 12.8 Hz, 3H), 3.71 (d, *J* = 12.8 Hz, 3H), 3.53 (q, *J* = 6.3 Hz, 1H), 3.11 (s, 4H), 2.39 (d, *J* = 4.1 Hz, 4H); ^13^C-NMR (150 MHz, CDCl_3_) δ 160.86, 158.19, 156.25, 152.75, 151.67, 150.99, 142.03, 130.90, 126.56, 125.77, 114.46, 113.15, 101.11, 98.73, 96.22, 95.21, 75.47, 59.25, 55.53, 55.18, 49.63, 48.66; IR(neat): 2951, 2877, 2834, 1592, 1569, 1538, 1519, 1484, 1453, 1418, 1316, 1248, 1212, 1161, 1028 cm^−1^.

### N*-(3-Methoxy-5–(8-azaspiro[4.5]decan-8-yl)phenyl)-7–(4-methoxyphenyl)-7*H*-pyrrolo[2,3-d]pyrimidin-2-amine (31)*

Following the procedure for **25, 20g** and **7** provided the title compound **31** (12%).

^1^H-NMR (600 MHz, CDCl_3_) δ 8.69 (s, 1H), 7.60 (dd, *J* = 6.8, 2.0 Hz, 2H), 7.19 (s, 1H), 7.14 (d, *J* = 3.6 Hz, 1H), 7.01–7.02 (m, 2H), 6.92 (s, 1H), 6.82 (d, *J* = 1.7 Hz, 1H), 6.53 (d, *J* = 3.6 Hz, 1H), 6.14 (d, *J* = 2.0 Hz, 1H), 3.86 (s, 3H), 3.70 (s, 3H), 3.06 (t, *J* = 5.6 Hz, 4H), 1.62–1.64 (m, 4H), 1.52 (t, *J* = 5.6 Hz, 4H), 1.43 (t, *J* = 7.1 Hz, 4H); ^13^C-NMR (150 MHz, CDCl_3_) δ 160.86, 158.22, 156.31, 153.41, 151.75, 150.95, 141.83, 130.89, 126.44, 125.61, 114.44, 113.12, 101.09, 99.02, 96.44, 94.79, 55.48, 55.17, 47.21, 40.73, 37.67, 37.32, 24.34; IR(neat): 2935, 1592, 1568, 1537, 1519, 1484, 1462, 1418, 1248, 1210, 1160, 1137, 1033, 831, 734 cm^−1^.

### N*-(3-Methoxy-5–(1-oxa-8-azaspiro[4.5]decan-8-yl)phenyl)-7–(4-methoxyphenyl)-7*H*-pyrrolo[2,3-d]pyrimidin-2-amine (32)*

Following the procedure for **25, 20h** and **7** provided the title compound **32** (31%).

^1^H-NMR (600 MHz, CDCl_3_) δ 8.69 (s, 1H), 7.60 (d, *J* = 8.6 Hz, 2H), 7.22 (s, 1H), 7.14 (d, *J* = 3.6 Hz, 1H), 7.02 (d, *J* = 8.6 Hz, 2H), 6.87 (d, *J* = 15.8 Hz, 2H), 6.53–6.53 (m, 1H), 6.14 (s, 1H), 3.86 (t, *J* = 6.7 Hz, 5H), 3.69 (s, 3H), 3.13–3.23 (m, 4H), 1.92–1.97 (m, 2H), 1.64–1.71 (m, 7H); ^13^C-NMR (150 MHz, CDCl_3_) δ 160.90, 158.23, 156.31, 152.87, 151.75, 150.97, 141.89, 130.89, 126.43, 125.61, 114.46, 113.15, 101.09, 99.20, 96.62, 94.93, 79.98, 66.70, 55.50, 55.17, 47.07, 36.26, 36.08, 25.48; IR(neat): 2938, 1592, 1568, 1538, 1519, 1484, 1462, 1418, 1347, 1300, 1248, 1209, 1161, 1133, 1034 cm^−1^.

### N*-(3-Methoxy-5–(4-(pyrrolidin-1-yl)piperidin-1-yl)phenyl)-7–(4-(trifluoromethoxy)phenyl)-7*H*-pyrrolo[2,3-d]pyrimidin-2-amine (33)*

Following the procedure for **25, 20b** and **12b** provided the title compound **33** (43%).

^1^H-NMR (600 MHz, CDCl_3_) δ 8.71 (s, 1H), 7.82 (dd, *J* = 7.0, 2.0 Hz, 2H), 7.36 (d, *J* = 8.6 Hz, 2H), 7.25 (s, 1H), 7.20 (d, *J* = 3.6 Hz, 1H), 6.92 (d, *J* = 1.5 Hz, 1H), 6.76 (s, 1H), 6.58 (d, *J* = 3.8 Hz, 1H), 6.16 (t, *J* = 2.0 Hz, 1H), 3.71 (s, 3H), 3.61 (d, *J* = 12.4 Hz, 2H), 2.68 (td, *J* = 12.2, 2.0 Hz, 2H), 2.60 (s, 4H), 2.10 (s, 1H), 1.93 (d, *J* = 12.4 Hz, 2H), 1.80 (s, 4H), 1.59–1.66 (m, 2H); ^13^C-NMR (150 MHz, CDCl_3_) δ 160.96, 156.48, 153.03, 151.78, 151.34, 147.14, 141.58, 136.43, 125.39, 124.98, 121.91, 121.85, 120.48 (q, *J* = 258.2 Hz), 113.34, 102.26, 99.29, 96.79, 95.60, 61.88, 55.14, 51.39, 48.47, 31.27, 23.27; IR(neat): 2952, 2800, 1596, 1572, 1540, 1511, 1483, 1461, 1415, 1379, 1355, 1256, 1206, 1161, 831 cm^−1^.

### N*-(3-Methoxy-5–(4-(pyrrolidin-1-yl)piperidin-1-yl)phenyl)-7-phenyl-7*H*-pyrrolo[2,3-d]pyrimidin-2-amine (34)*

Following the procedure for **25, 20 b** and **12a** provided the title compound **34** (17%).

^1^H-NMR (600 MHz, CDCl_3_) δ 8.71 (s, 1H), 7.75 (d, *J* = 7.6 Hz, 2H), 7.50–7.52 (m, 2H), 7.37 (t, *J* = 7.4 Hz, 1H), 7.28 (s, 1H), 7.23 (d, *J* = 3.7 Hz, 1H), 6.88 (dd, *J* = 21.9, 1.7 Hz, 2H), 6.57 (d, *J* = 3.7 Hz, 1H), 6.13 (t, *J* = 2.0 Hz, 1H), 3.69 (s, 3H), 3.60 (d, *J* = 12.6 Hz, 2H), 2.75 (s, 4H), 2.63 (td, *J* = 12.3, 1.9 Hz, 2H), 2.29 (s, 1H), 1.95 (d, *J* = 12.4 Hz, 2H), 1.88 (s, 4H), 1.68–1.70 (m, 2H); ^13^C-NMR (150 MHz, CDCl_3_) δ 160.87, 156.30, 152.85, 151.67, 151.10, 141.84, 137.84, 129.34, 126.56, 126.02, 123.98, 113.41, 101.65, 99.26, 96.87, 95.27, 61.93, 55.23, 51.17, 48.43, 30.50, 23.32; IR(neat): 2956, 1593, 1568, 1538, 1517, 1500, 1482, 1460, 1416, 1353, 1270, 1208, 1157, 1070, 759 cm^−1^.

### N*-(3-Methoxy-5–(4-(pyrrolidin-1-yl)piperidin-1-yl)phenyl)-7-(thiazol-2-yl)-7*H*-pyrrolo[2,3-d]pyrimidin-2-amine (35)*

Following the procedure for **25, 20 b** and **8** provided the title compound **35** (11%).

^1^H-NMR (600 MHz, DMSO-D_6_) δ 9.56 (s, 1H), 8.84 (s, 1H), 7.97 (d, *J* = 3.8 Hz, 1H), 7.93–7.76 (1H), 7.70 (d, *J* = 3.4 Hz, 1H), 7.34–7.09 (1H), 6.98 (s, 1H), 6.78 (d, *J* = 3.8 Hz, 1H), 6.16 (s, 1H), 3.74 (s, 5H), 3.33 (s, 5H), 2.68 (s, 2H), 1.96 (d, *J* = 120.2 Hz, 9H); ^13^C-NMR (150 MHz, DMSO-D_6_) δ 160.20, 156.38, 154.85, 151.79, 150.21, 141.47, 140.53, 138.59, 123.16, 116.82, 112.72, 103.86, 100.17, 96.47, 96.00, 60.94, 54.93, 50.49, 47.59, 40.02, 39.97, 39.93, 39.85, 39.71, 39.57, 39.43, 39.29, 39.15, 39.01, 38.89, 28.90, 22.60; IR(neat): 3403, 2917, 2360, 1594, 1576, 1542, 1523, 1508, 1483, 1452, 1409, 1354, 1221, 1157, 764 cm^1^.

### *(1r,4r)-4–(2-((3-Methoxy-5–(4-(pyrrolidin-1-yl)piperidin-1-yl)phenyl)amino)-7*H*-pyrrolo[2,3-d]pyrimidin-7-yl)cyclohexanol (36)*

Following the procedure for **25, 20 b** and **12c** provided the title compound **36** (16%).

^1^H-NMR (600 MHz, DMSO-D_6_) δ 9.21 (s, 1H), 8.65 (s, 1H), 7.36 (d, *J* = 3.4 Hz, 1H), 7.33 (s, 1H), 6.97 (s, 1H), 6.43 (d, *J* = 3.4 Hz, 1H), 6.09 (s, 1H), 4.54 (q, *J* = 5.0 Hz, 1H), 3.73 (s, 3H), 3.67 (d, *J* = 9.0 Hz, 2H), 3.58 (s, 1H), 2.74 (t, *J* = 11.0 Hz, 5H), 2.00 (t, *J* = 10.7 Hz, 4H), 1.88–1.92 (m, 4H), 1.74 (s, 4H), 1.58 (s, 2H), 1.32–1.37 (m, 2H), 1.23 (s, 1H); ^13^C-NMR (150 MHz, DMSO-D6) δ 160.06, 155.47, 150.42, 150.34, 142.64, 123.55, 111.92, 99.49, 98.35, 95.18, 94.75, 67.61, 60.89, 54.62, 51.76, 50.70, 47.48, 34.47, 30.25, 22.75; IR(neat): 3377, 2936, 1593, 1570, 1537, 1487, 1453, 1422, 1381, 1199, 1158, 1070, 1026 cm^−1^.

### Kinase assay

All kinase assays were carried out at Km ATP by Eurofins Discovery's Kinase Screening and Profiling services (France).

### Cell culture

MKN28 cells were obtained from the Korea Institute of Science and Technology (KIST). Cells were cultured in Roswell Park Memorial Institute (RPMI) 1640 medium containing 10% fetal bovine serum and 1% penicillin/streptomycin at 37 °C with 5% CO_2_ under a humidified atmosphere.

### Western blot

MKN 28 cells (500,000 cells/2 ml) were seeded in each well of 6-well plates and incubated for 24 h. Then, the cells were treated with 20 µL of DMSO stock solution of the corresponding compounds and incubated for a further 1.5 h. After that, the cells were washed with cold Dulbecco's Phosphate-Buffered Saline (DPBS) twice and lysed with RIPA buffer supplemented with protease inhibitor and phosphatase inhibitor cocktails on ice. Equal amounts of protein samples were boiled with 5× SDS-PAGE loading buffer and separated by 10% SDS-PAGE gels, then transferred onto polyvinylidene difluoride (PVDF) membranes. The membranes were blocked with 5% BSA in 1× TBST (1× TBS with 0.1% Tween-20) for 1 h and incubated at 4 °C overnight with a 1:1000 dilution of the following primary antibodies in blocking buffer: pMer (Tyr749 + 753 + 754, ab14921), Mer (CST, # 4319), and ß-actin (SC, # SC-47778). After three washes with 1× TBST, the membranes were incubated with a 1:2000 dilution of the following secondary antibodies at RT for 1 h followed by extensive washing three times. Secondary antibodies: anti-rabbit IgG, HRP-linked antibody (CST, # 7074S); anti-mouse IgG, HRP-linked antibody (CST, # 7076S). Antibody binding was visualised by an enhanced chemiluminescent (ECL) system (Bio-Rad, Clarity Western ECL Substrate, # 1705061) and VILBER FUSION SOLO X. Antibodies were purchased from Abcam, Cell Signalling Technology (CST), and Santa Cruz Biotechnology (SC). ß-actin was used as a loading control.

## Results and discussion

To find the novel Mer and Axl inhibitors, we screened our in-house chemical library for the TAM family. As shown in Table 1, 7-aryl pyrrolopyrimidines **2** and **3** were found to be hits, with IC_50_ of 39 nM and 95 nM against Mer, respectively, and little activity for Tyro3. 3-phenyl aniline **4** and piperazine **5** did not have activity against the TAM family. Thus, derivatives of compound **2** were synthesised to explore the structure-activity relationship.

**Table 1.** Activity for TAM kinases of methoxy phenyl pyrrolopyrimidines.


Compound	R1	IC50 (nM)		% Inhibition at 1 µM
		MERTK	AXL	TYRO3
**2**		39	1240	38
**3**		95	751	44
**4**		>3000	>3000	12
**5**		>1000	>1000	0
**UNC569**		3	420	ND^a^

Data are mean values. ^a^ND: not determined.

**Scheme 1.**
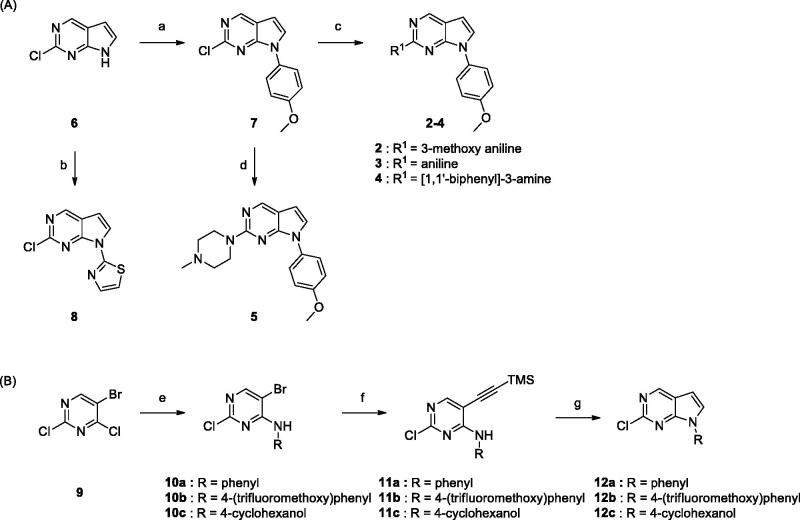
Reagents and conditions: (a) (OH)_2_B-Ar, Cu(OAc)_2_, pyridine, 4 Å MS, CH_2_Cl_2_, rt, 6–24 h; (b) 2-bromothiazole, CuI, K_3_PO_4_, 1,2-*trans*-cyclohexanediamine, THF, 110 °C, 24 h; (c) BINAP, Pd_2_(dba)_3_, Cs_2_CO_3_, dioxane, 100 °C, 8 h; (d) HCl, *i*-PrOH, MW 160 °C, 1 h; (e) 4-(trifluoromethoxy)aniline, TEA, *i*-PrOH; (f) ethynyltrimethylsilane, Pd(PPh_3_)_2_, CuI, TEA, toluene, 80 °C, 4 h; (g) TBAF, THF, 60 °C, 4 h.

**Scheme 2.**
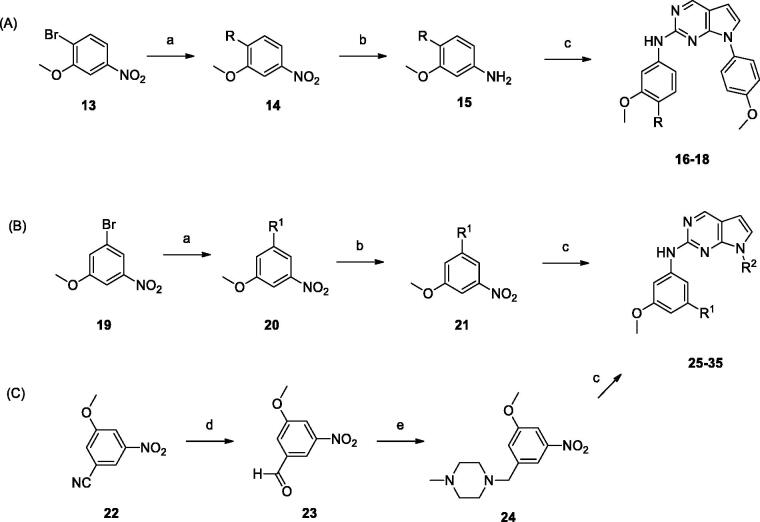
Reagents and conditions: (a) amines, Pd_2_(dba)_3_, BINAP, Toluene, Cs_2_CO_3,_ 100 °C, 6 − 24 h; (b) H_2_, Pd/C, MeOH, rt, 3 − 9 h; (c) **7, 8,** or **12**, HCl, *i*-PrOH, MW 160 °C, 1 h; (d) DIBAL, toluene, 0 °C, 3 h; (e) 1-methylpiperazine, NaBH(OAc)_3_, AcOH, DCE, rt, 12 h.

The synthetic routes for the derivatives are illustrated in Schemes 1 and 2. As shown in Scheme 1, 2-chloro-pyrrolo[2,3-d]pyrimidine intermediates with various substituents were prepared by reacting commercially available 2-chloro-7*H*-pyrrolo[2,3-d]pyrimidine **6** with boronic acids using the Chan–Lam coupling reaction^14^. Thiazole **8** was synthesised by the Ullmann coupling reaction^15^. For the synthesis of further derivatives, intermediates **10a–c** were synthesised by nucleophilic substitution of amine with **9**. Sonogashira coupling of **10a–c** with TMS acetylene followed by intramolecular cyclisation gave the desired intermediates **12a**–**c**.

Commercially available 1-bromo-2-methoxy-4-nitrobenzene **13** was coupled with amines by the Buchwald–Hartwig coupling reaction. The resulting alkylamino compound **14** was hydrogenated to yield aniline **15**, followed by coupling with 2-chloropyrrolopyrimidines (**7**, **8,** and **12a-c**) to give the desired compounds **16–18.** Synthesis of *meta*-substituted derivatives started from 3-bromo-5-methoxy nitrobenzene. Compounds **22–32** were obtained using the same synthetic methods as those used to prepare **16–18**. Derivative **23** was synthesised from 3-cyano-5-methoxy nitrobenzene, as outlined in Scheme 2(c).

Introduction of an alkyl amino group at the *para*-position resulted in an improvement of activity over compound **2** with weak activity against Tyro3 (Table 2). *N*-Methyl piperazinyl derivative **16** showed 2.6-fold and 12-fold decreased IC_50_ for Mer and Axl, respectively, compared to **2**. Compound **17** also had IC_50_ of 10 nM for Mer and approximately 200 nM for Axl without considerable inhibition of Tyro3. Dimethylaminopyrrolidine **18** showed a slight decrease in activity against Mer and improved activity against Axl.

Next, the effects of *meta*-substitution were explored. Interestingly, substitution of *N*-methyl piperazine (**25**) at the *meta*-position (R^3^) led to great potency for Mer with an IC_50_ of 2 nM. The one-carbon extended N-methyl piperazine derivative **26** was also highly potent, but it showed relatively high activity for Tyro3 compared to the other derivatives. 4-Pyrrolidinyl piperidine derivative **27** was also equipotent to the known compound (**UNC569)** for Mer and Axl but still showed weak activity against Tyro3. Methylated derivative **28** displayed 2–3-fold weaker activity than **27**. 3-Pyrrolidinyl piperidine **29** had moderate activity for Mer and Axl whilst 4-oxetanyl piperazine **27** showed decreased activity for Axl. Interestingly, the introduction of an azaspirodecanyl group (**31**) led to complete loss of activity for all TAMs. However, the insertion of oxygen (**32**) resulted in the recovery of activity similar to **30**. These data suggest that a heteroatom at an appropriate distance from the aniline ring might be necessary to achieve good activity for Mer and Axl. A docking study was carried out to further understand the binding mode of the described compounds.

**Figure 2.**
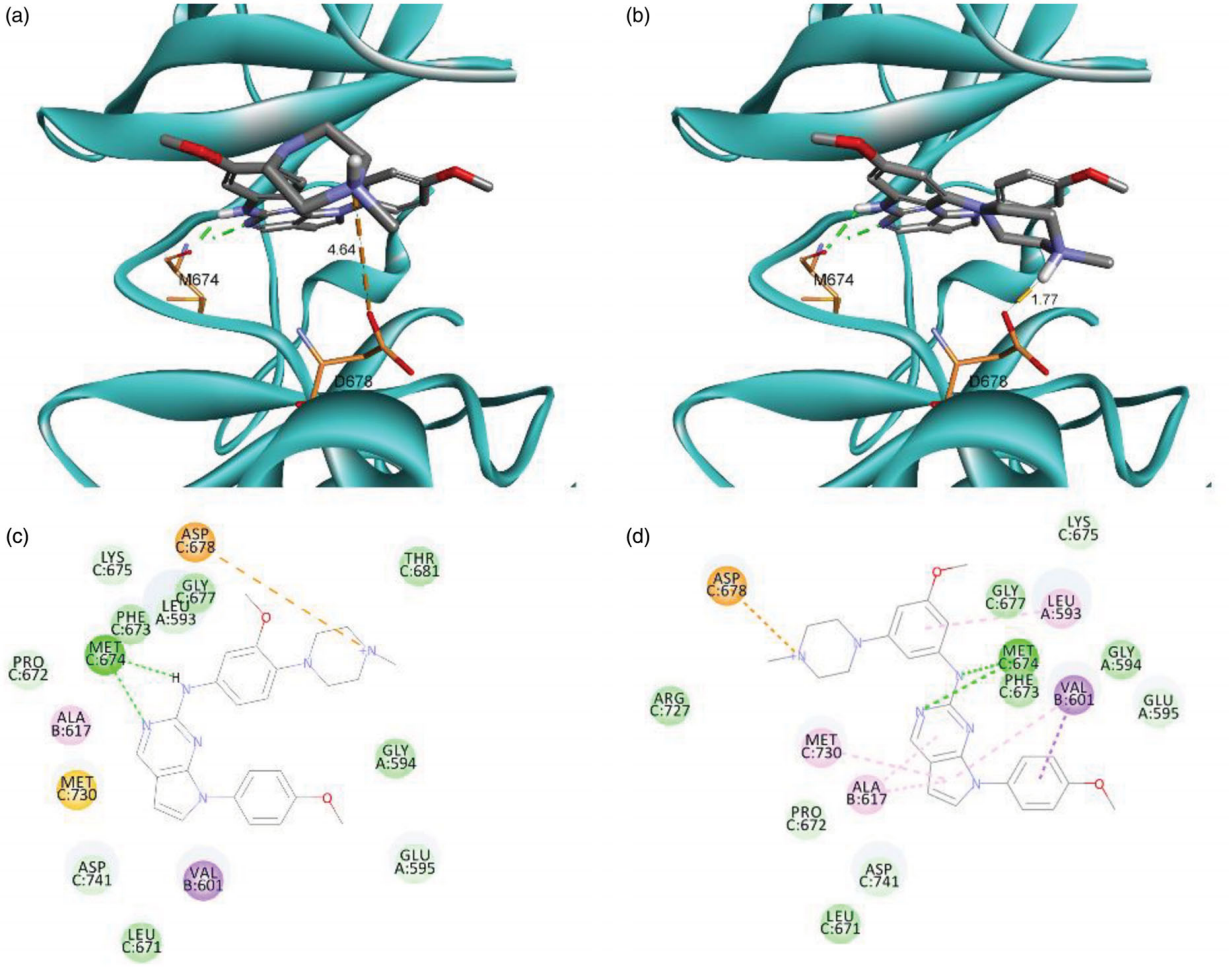
Predicted docking orientation of **16** and **25** with the Mer kinase domain (PDB ID: 3TCP). Docking mode of (a) **16** and (b) **25** with Mer. 2 D-interaction diagram of the binding model of (c) **16** and (d) **25**. Estimated binding energies were −7.52 kcal/mol and −8.26 kcal/mol for **16** and **25**, respectively. Hydrogen bonds and a salt bridge between the ligand and the backbone are shown in dashed lines. The docking study was performed by AutoDock Vina.

As shown in Figure 2, the docking model showed that N^1^ and NH of **16** and **25** interact with MET674 of the hinge region through hydrogen bonding, and the nitrogen atom in the piperazine moiety forms a salt bridge with ASP678. The distances between ASP678 and the nitrogen atom in the piperazine moiety of *para*-derivative **16** and *meta*-derivative **25** were calculated as 4.64 Å and 1.77 Å, respectively. This suggests that the piperazine moiety at the *meta*-position could be placed closer to ASP678 than when it is at the *para*-position, which may induce stronger binding of **25** than that of **16**. In addition, this docking model provided a reasonable explanation for the complete loss of activity of **31**, which cannot interact with ASP678 owing to the absence of a nitrogen atom. The addition of an oxygen atom at the spiro-ring in **32** led to recovered activity, which also supported the docking model. These data indicate that the formation of a salt bridge with ASP678 is important for retaining activity against TAM family kinases.

Next, a brief structure-activity relationship investigation was carried out for R^4^, as shown in Table 3. According to the predicted docking model for Mer, it was postulated that R^4^ as a 4-methoxyphenyl group is positioned in the hydrophobic pocket and is involved in a π–alkyl interaction with Val601. As expected, trifluoromethyl phenyl compound **33** and phenyl compound **34** showed excellent activity for Mer and Axl. Thiazole derivative **35** also retained activity, although its activity was less than those of **33** and **34**. However, the introduction of a cycloalkyl group, a *trans*-4-hydroxycyclohexyl group, in **36** significantly decreased the activity for Mer and Axl. This suggests that the described compounds may interact with Mer in a different manner than **UNC569**. The data indicate that *N*^7^-substituents with an aromatic group may be suitable to bind Mer or Axl.

**Table 2.** Activities of 3-methoxy aniline derivatives of compound 3.


Compound	R2	R3	IC50 (nM)		% Inhibition at 1 µM
			MER	AXL	TYRO3
**16**		H	15	104	27
**17**		H	10	199	37
**18**		H	46	175	30
**25**	H		2	17	86
**26**	H		4	18	92
**27**	H		2	16	40
**28**	H		7	32	72
**29**	H		31	31	49
**30**	H		36	212	39
**31**	H		>3000	>3000	4
**32**	H		40	288	0
**UNC569**			3	420	ND^a^

^a^ND: not determined.

**Table 3.** Inhibitory activity of R^4^ derivatives.
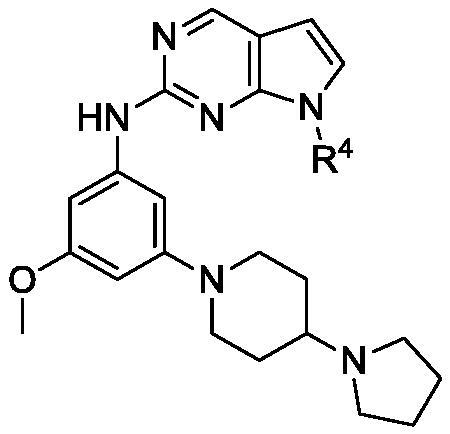

Compound	R^4^	IC_50_ (nM)		%Inhibition at 1 µM
		MER	AXL	TYRO3
**33**		9	57	23
**34**		17	48	35
**35**		64	247	16
**36**		484	2018	55
**UNC569**		3	420	ND^a^

^a^ND: not determined.

**Figure 3.**
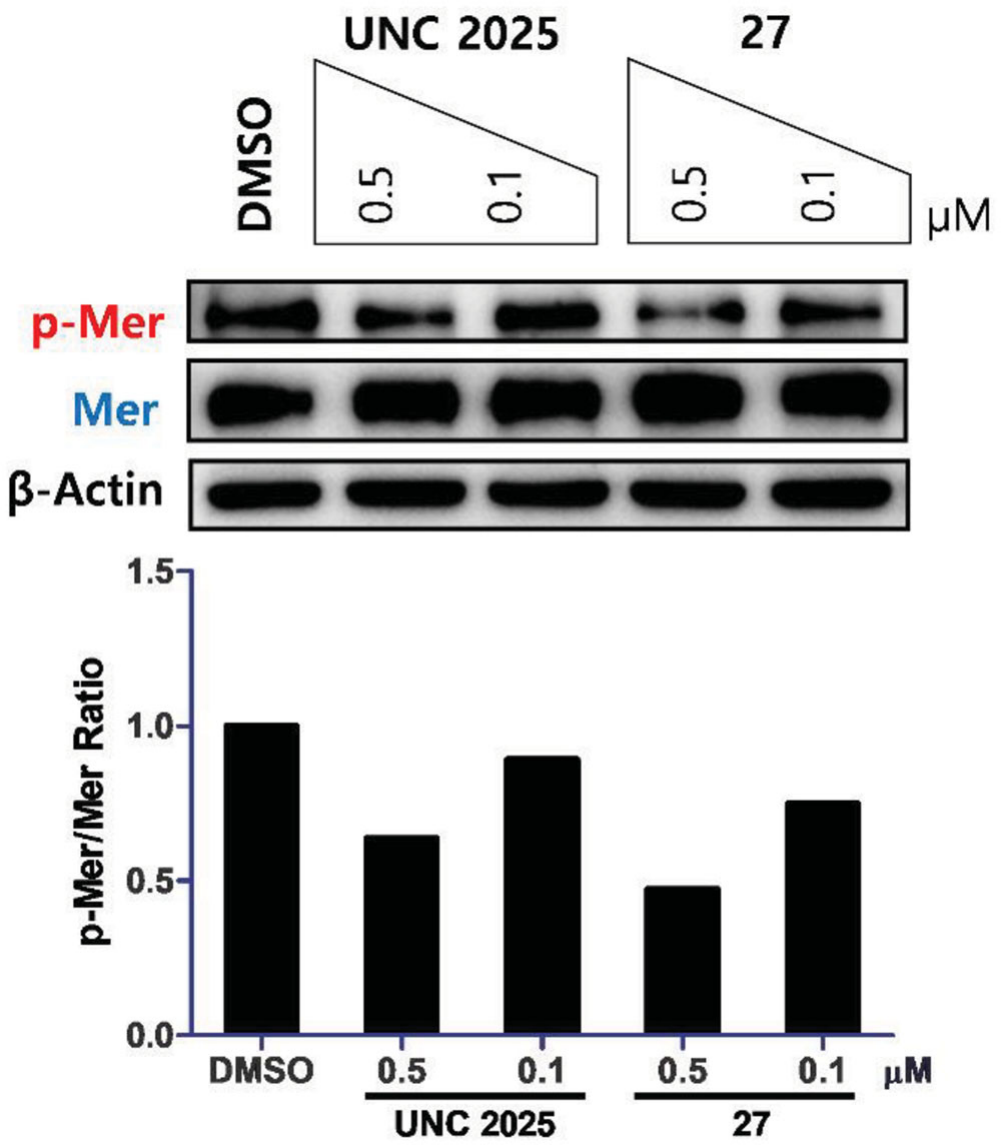
Inhibitory effect of **27** on Mer phosphorylation in a Mer-overexpressed human gastric cancer cell line, MKN28. Cells were treated with the indicated compounds at 0.5 and 0.1 μM for 1.5 h. **UNC2025** and β-actin were used as a positive control and a loading control, respectively. Western blot analysis for phosphorylated and total Mer from a representative experiment is shown. Bar graph represents the relative intensities of the total and phosphorylated Mer as determined by band densitometry using image analysis software.

To determine the inhibitory activity of compounds on Mer phosphorylation in cells, western blot analysis was carried out. A representative compound **27** was used to treat a Mer-overexpressed human gastric cancer cell line, MKN28. A potent Mer inhibitor, **UNC2025**, was used as a positive control (Figure 3). Compound **27** showed a better effect on blocking phosphorylation than **UNC2025** at the indicated concentrations.

In summary, we report here the discovery of 7-aryl-2-anilino-pyrrolopyrimidine derivatives as potent inhibitors of Axl and Mer kinases without considerable inhibition of Tyro3. The most potent compound **27** had IC_50_ values of 2 nM and 16 nM for Mer and Axl, respectively, but just 40% inhibition of Tyro3 at 1 µM. In addition, compound **27** exhibited considerable inhibition for Mer phosphorylation in a cancer cell line. Structure-activity relationship and docking studies showed that forming a salt bridge and an aromatic group at the N^7^ position are essential for its Axl and Mer kinase inhibition activity. This work could provide useful information for the molecular design of Axl/Mer kinase inhibitors.
